# Current status and potential application of navigated transcranial magnetic stimulation in neurosurgery: a literature review

**DOI:** 10.1186/s41016-019-0159-6

**Published:** 2019-05-21

**Authors:** Xiaojing Fang, Meige Liu, Changyu Lu, Yuanli Zhao, Xianzeng Liu

**Affiliations:** 1 Department of Neurology, Peking University International Hospital, 1 Life Science St, Changping District, Beijing, 102206 China; 2 Neurosurgery Center, Beijing Tiantan Hospital, Capital Medical University, Beijing, 100070 China; 3 Department of Neurosurgery, Peking University International Hospital, Beijing, 102206 China; 4 Department of Neurology, Peking University People’s Hospital, Beijing, 100044 China

**Keywords:** Transcranial magnetic stimulation, Motor mapping, Speech mapping, Neurosurgery

## Abstract

Transcranial magnetic stimulation (TMS) is a noninvasive neurophysiologic technique that can stimulate the human brain. Positioning of the coil was often performed based merely on external landmarks on the head, meaning that the anatomical target in the cortex remains inaccurate. Navigated transcranial magnetic stimulation (nTMS) combines a frameless stereotactic navigational system and TMS coil and can provide a highly accurate delivery of TMS pulses with the guidance of imaging. Therefore, many novel utilities for TMS could be explored due to the ability of precise localization. Many studies have been published, which indicate nTMS enables presurgical functional mapping. This review aimed to provide a comprehensive literature review on nTMS, especially the principles and clinical applications of nTMS. All articles in PubMed with keywords of “motor mapping,” “presurgical mapping,” “navigated transcranial magnetic stimulation,” and “language mapping” published from 2000 to 2018 were included in the study. Frequently cited publications before 2000 were also included. The most valuable published original and review articles related to our objective were selected. Motor mapping of nTMS is validated to be a trustful tool to recognize functional areas belonging to both normal and lesioned primary motor cortex. It can offer reliable mapping of speech and motor regions at cortex prior to operation and has comparable accuracy as direct electrical cortical stimulation. nTMS is a powerful tool for mapping of motor and linguistic function prior to operation, has high application value in neurosurgery and the treatment of neurological and psychiatric diseases, and has gained increasing acceptance in neurosurgical centers across the world.

## Main text

Since the introduction for stimulating the human motor cortex by transcranial magnetic stimulation (TMS) in 1985 [1], TMS has been applied in studying the processing of cortex information and treating psychiatric and neurologic diseases [2–6]. However, how to precisely locate the magnetic coil at cortical regions remains a major obstacle in relevant investigations. In most cases, the anatomic features of involved subjects were not taken into consideration in TMS studies, so it is very difficult to reach the intended area accurately [7].

In the past decades, a combination of optically tracked stereotactic navigation systems and TMS technology was developed. This system can form a picture of the stimulation sites through the three dimensions (3D) rebuilt MRI data of the subjects’ brain, which was therefore named as navigated transcranial magnetic stimulation (nTMS) [8, 9]. NTMS has been tested for mapping of the motor cortex prior to operation and is widely accepted by neurosurgeons [10–12]. Motor mapping by nTMS correlate well with mapping of direct electrical stimulation (DES) during surgery, especially when compared to other preoperative mapping methods such as functional magnetic resonance imaging (fMRI) or magnetoencephalography [13, 14]. In recent years, nTMS has been widely used in motor mapping, and more evidence shows that it is beneficial for patients with brain tumor. Many studies observed better outcomes and higher survival rates in brain tumor patients who use nTMS before surgery, thus expanding the initial role of nTMS as a mere preoperative planning tool [15, 16].

We review the basic principles and clinical applications of nTMS, protocols for motor and language mapping, comparison between nTMS and other preoperative mapping modalities, and future applications of nTMS, so as to improve the understanding and clinical application of nTMS.

### Electric-field navigation and line navigation

Many techniques can be applied to the navigation of TMS. They can be divided into two groups, namely, line-navigated transcranial magnetic stimulation (Ln-TMS) and electric-field navigated transcranial magnetic stimulation (En-TMS). Ln-TMS exhibits the location of the coil while not showing coil angulation in relation to the patient’s head. The stimulation spot is assumed to be on the line that passes through the geometric center of the coil and is perpendicular to the coil surface [17], and if the coil is not perfectly tangential with respect to the skull, the target would be imprecise [18]. En-TMS can calculate the electric field on the cortex while stimulating. It can visualize and calculate the electric field with its dose and orientation, as well as optimize the coil positioning continuously. The resolution of En-TMS reaches 2 mm [11, 16]. At present, only the En-TMS system could be used for tumor-patient diagnostics [11–13, 19–21]. As what were already clarified for En-TMS, more investigations using intraoperative DES are needed to determine whether Ln-TMS can produce motor mapping with high specificity and accuracy in neurosurgical operations.

### Motor mapping and speech mapping protocols

**Fig. 1:**
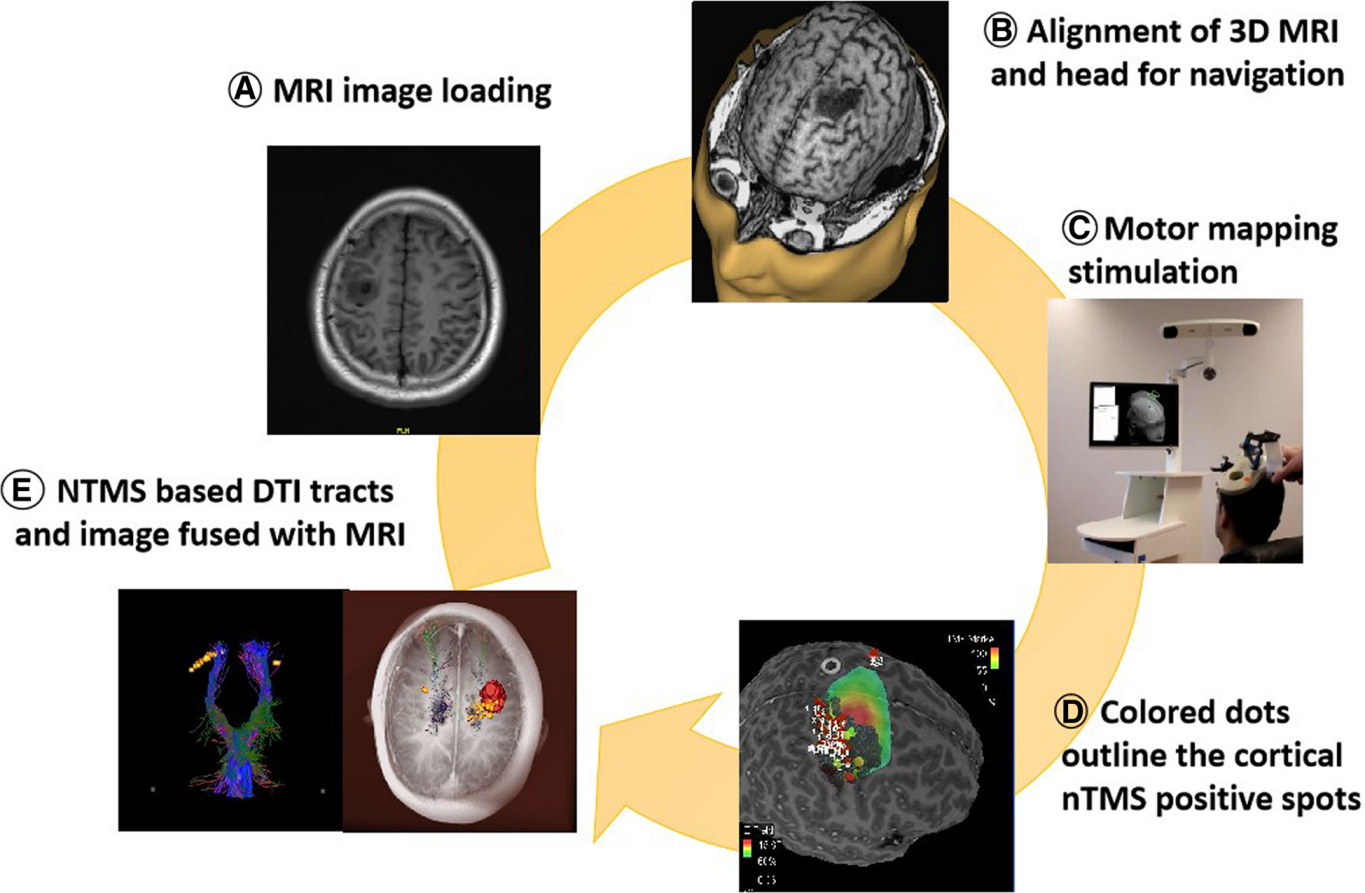
The procedure of nTMS motor mapping and image of nTMS-based DTI tracts. (A) Upload T1-weighted image. (B) Overlay of the 3D head model derived from MRI imaging and the actual patient head by means of surface registration to enable real-time neuronavigation. (C) Motor mapping stimulation. (D) Mark the nTMS positive spots on the cortex. (E) Left, nTMS-based DTI tracts: cortical nTMS positive spots (yellow) and nTMS-based DTI tracts (blue); Right, image fused with MRI: tumor (red), cortical nTMS positive spots (yellow), and nTMS-based DTI tracts (blue). DTI diffusion tensor imaging, MR magnetic resonance, nTMS navigated transcranial magnetic stimulation

Preparation for the session:

Upload individual MRI data and use them to generate a 3D head model. Match the 3D head model with the patients’ head (Fig. 1).

Hot spot identification:

The rapid changes of transcranial magnetic field produce electric current at a specific region at the cortex, leading to a corresponding motor evoked potential (MEP) [22]. Hot spot identification is the search for the cortical sites producing maximum MEPs [23, 24].

RMT (resting motor threshold) determination:

RMT refers to the lowest intensity of TMS which is able to elicit a 50-μV MEP in a relaxed small hand muscle in 5 out of 10 stimulations. The target muscle should be at rest, and monitoring of muscle activity is critical during the determination of the RMT [25].

Set the stimulate mode to single pulse TMS and set the output of stimulator to 110% RMT when doing motor mapping. Stimulate the central sulcus, precentral sulcus, postcentral sulcus, and precentral gyrus with a 2–3-mm interval [26]. Mark the nTMS positive spots on the cortex (Fig. 1).

Most language mapping protocols use object naming and navigated repetitive TMS (nrTMS) combined with video recording of the behavioral results. The stimulate strength is 100–120% of the RMT for the hand muscles. The stimulate spot is not restrained to conventional regions stated by the Geschwind’s language model [27, 28] The corresponding nrTMS locations in the parcellated cortex are marked as language-related and are tagged by the observed error type [29, 30].

### Safety issue

It has been proved that nTMS is safe in both normal subjects and patients with intracranial lesions. The only absolute contraindication of nTMS is the presence of metal or electronic devices, such as cochlear implants, pulse generators, and medical pumps, near the coil stimulation site, which are at risk of being destroyed [31]. A cohort study involving 733 patients showed that nTMS was well tolerated in motor mapping, with only 6% of patients feeling unwell. In language mapping, as nrTMS was used, 93% of the patients felt uncomfortable and 70% of the patients felt pain. The discomfort of repetitive stimulation was mainly caused by contraction of the temporalis muscle and orbicularis oculi muscle and direct stimulation of trigeminal nerve sensory branches [32, 33]. There are few reports on seizure induced by single-pulse TMS in patients with neurological diseases; therefore, it should be noted that patients with cerebral lesions might still have a high risk of seizure induced by TMS [34].

### Comparison between nTMS and direct electrical cortical stimulation

DES has always been accepted as the gold standard for motor mapping and language mapping [35]. It was reported that motor activation points detected by nTMS were closely associated with those found in DES [36, 37]. One meta-analysis reviewed six studies with a total of 81 patients and summarized that the mean distance of motor cortex identified on DES and nTMS was 6.18 mm [37]. Two other studies mapped the motor cortex in patients with growing malignant and large tumors, and they found that the distances of DES and nTMS were 10.5 mm and 7.83 mm, respectively [11, 12]. Pavia reported a distance of approximately 4 mm in a group of patients with low-grade gliomas. This may be explained by deficiency of accompanied edema and the relative small size of the lesions [38].

Language mapping conducted by NrTMS has shown close association with DES, and compared with DES, nTMS has a sensitivity value of 90%, specificity of 98%, negative predictive value of 99%, and positive predictive value of 69% [31, 39–41]. NrTMS can be completed before operation, which is of great significance to the formulation of surgical plans. Preoperative localization of language function using nTMS is helpful to reduce the incision and improve the accuracy of intraoperative cortical electrical stimulation; therefore, functioning language regions under intraoperative examination should be expanded and not just limited to known anatomic areas [42]. Language mapping by nrTMS can help identify language-eloquent areas for patients not eligible for awake surgery and can be used for smaller craniotomies and focused intraoperative DES, increasing efficiency and safety [31].

NTMS mapping is highly reliable and can significantly shorten intraoperative DES mapping, but it still can not replace DES or interoperative monitoring (IOM) completely. On the contrary, nTMS serves as a valuable supplement for IOM but not a competing modality [43]. They should be used together to optimize the patient’s selection and approach planning, accordingly accelerating the surgery and having better oncological and functional outcomes [15, 44].

### nTMS and fMRI

At present, fMRI is the most frequently applied imaging methods for motor and linguistic function localization before operation. Compared with fMRI, nTMS imaging has better time resolution and does not require complex post-processing analysis. It also can be applied to patients with claustrophobia who can not complete the MRI examination [45]. The advantages and disadvantages of nTMS compared to fMRI and DES are shown in Table 1.

**Table 1:** Comparison among nTMS, DES, and fMRI

	nTMS	DES	fMRI
Safety issue	Safe, noninvasive	Invasive	Safe, noninvasive
Accuracy	High accuracy	Gold standard for mapping of motor and language	Accuracy is affected by patient’s cooperation Less reliable for motor mapping in patients with brain tumor
Maneuverability	Simple Do not need patient’s cooperation	Complex	Require complex post-processing analysis Need patient’s cooperation
Expenses	Low	High	Low
Level of comfort	Well tolerated in motor mapping A little uncomfortable in language mapping	Invasive	Comfortable but can not be applied to claustrophobic patients

Researches showed that fMRI was less reliable for motor mapping in patients with brain tumor, especially in the regions near the lesion [46, 47]. NTMS showed a better tracking efficiency than fMRI in cases that the cortical tract origin was very close to a brain tumor probably because the tumor influenced the neurovascular coupling. When blood oxygenated level-dependent signal physiology was altered, nTMS appeared to be a better option [48].

Scientists have reported the combined use of nTMS and diffusion tensor imaging fiber tracking (DTI-FT) in the cortical spinal tract (CST) [48]. The DTI-FT based on nTMS is proved to be a more dependable and precise tool to reconstruct the CST compared with the standard anatomical tractography. The functional and anatomical data acquired from the somatotopic reconstruction make it possible to assess the spatial correlation between the motor fibers and the lesion and improve the evaluation of the risks of tumor resection. In addition, the nTMS-based DTI-FT of the CST is able to use during surgery as a guide for orienting DES and lesion resection. Recent study showed that nTMS mapping especially nTMS-based DTI-FT can be served as a suitable surgical technique for motor-eloquent lesions and is likely to promote the risk/benefit analysis, resection extent, and outcomes [48–50].

The identified language-positive nrTMS spots can also be used as regions of interest (ROIs) for nTMS-based DTI-FT. Sollmann et al. did preoperative nrTMS language mapping in 40 patients and defined each error categories separately as a ROI, which was used for function-specific nTMS-based DTI-FT. Their results suggested that using different error categories as ROIs could result in better guidance during operation [51].

### Clinical use of nTMS in neurosurgery

It is a great challenge for neurosurgeons and patients to perform a brain tumor resection located at or near the motor eloquent area. In brain tumor surgery, preserving neurological function and taking maximal resection are major principles, and neurosurgeons frequently encounter a dilemma between conservation of motor function and completeness of resection. Therefore, in order to reduce motor deficits, preoperative functional localization of nTMS is necessary. Intracranial lesions, such as ischemia or tumors, are likely to result in the displacement and remodeling of motor and speech areas. NTMS can accurately determine the displaced functional areas, thus providing assistance for surgical planning [52]. It is reported that nTMS not just affects indication and planning in surgery but also results in a higher rate of gross total resection and a lower rate of surgery-related paresis. Expanding indications for surgery and the extent of resection according to nTMS results enables more patients to undergo surgery and lead to better outcomes and high survival rates [44].

Krieg compared the surgical outcome of patients with motor eloquent metastatic lesions who received preoperative nTMS-based motor mapping with those that did not. Patients receiving nTMS had a lower rate of residual tumor, smaller craniotomies, shorter operation time, and decreased surgery-related paresis [53]. Krieg comprehensively studied the impact of preoperative motor mapping by nTMS on different clinical outcome parameters within a homogeneous cohort of high-grade glioma patients. The results demonstrated that patients who underwent nTMS preoperative mapping had smaller craniotomies and less residual tumor tissues [54]. Picht used nTMS in a group of patients with gliomas and found nTMS mapping could change the therapy planning into early and more extensive resection. The median change of tumor volume from baseline to 1 year was − 83% in the nTMS group, but + 12% in the comparison group [55].

Functional mapping with nTMS is available not only in tumorous brain lesions, but also within hypervascularized cortical areas [56]. Many studies had shown that nTMS can serve as a powerful tool to schedule planning prior to surgery for patients with arteriovenous malformation and cavernous angiomas, and it can also help optimize treatment planning [36, 57].

The mapping based on nTMS can visualize the language network more efficiently and can also detect cortical plasticity induced by an intra-hemispheric tumor-induced cortical plasticity. It can be used to formulate a tailored surgical plan which could preserve language function after the surgery. This tool could play a supplementary role for neurosurgeons in dealing with patients with potential language-eloquent tumors, but not applicable for awake surgery [58].

Besides surgical planning, the nTMS data and nTMS-based tractography are far beyond its current application. Schwendner implemented nTMS motor mapping in patients with intracranial metastases during routine radiotherapy. The results showed that it can significantly reduce the dose applied to the motor cortex while not affecting the treatment doses for the planning target volume [59].

NTMS can also be used in the prediction of recovery of paralysis. One study demonstrates that nTMS results before and after surgery have the potential to predict the motor function recovery after glioma resection. At 1 week after surgery, positive MEPs induced by nTMS signify a better recovery from postsurgical neurological deficits [60]. Peters examined the association of MEPs with lower extremities outcomes in a well-defined cohort of chronic, stable stroke survivors. They found MEP latency appears to be an indicator of lower extremities impairment and gait [61].

### Broad application of nTMS 

Single-pulse TMS can provide insight into the excitability and integrity of the CST [62], and paired-pulse TMS can provide insight into the excitability and integrity of corticocortical connections [63]. Common evaluation parameters of MEP include amplitude, latency, and central motor conduction time [64]. In clinical application, location and strength of the electric field could result in alterations in MEP amplitudes. Schmidt et al. demonstrated that the variability of cortical spinal excitability (CSE) is decreased by controlling the physical parameters via navigation. NTMS can help accurately identify and sustain nTMS coil’s location to the targeted muscle’s representation on the cortex, and this allows increasing the accuracy of nTMS motor mapping and the assessing of CSE [65].

Studies have used nTMS to evaluate the function of upper motor neurons in patients with amyotrophic lateral sclerosis (ALS). The RMT was significantly higher in ALS patients, and the mean motor mapping areas were smaller in patients with ALS than in controls. They found nTMS is a promising method for assessing upper motor neuron function in ALS and may clarify the pathogenic process of neurodegeneration and help establish novel diagnostic and prognostic models [66].

In recent years, preservation of language and motor function had been prioritized over other necessary functions. Several centers also used TMS to map further essential brain functions [5, 67]. Since TMS has been applied for mapping of neuropsychological cortical function, it seems feasible to map different neuropsychological functions more accurately by nTMS. It is reported that nTMS is available to accurately map calculation function at cortex [68] and cortical face processing function in healthy subjects [69].

An international investigation obtained level A evidence in determining the efficacy of repetitive TMS (rTMS) on neuropathic pain [70, 71]. In most rTMS studies, stimulating the motor cortex at the opposite side of the aching side showed analgesic effects, regardless of the location of the pain [72, 73]. Few studies reported using image-guided navigated systems to identify motor cortex in pain treatment by rTMS [71, 74]. An image-guided navigated procedure might provide a customized solution for each patient receiving rTMS pain treatment. In one study, the analgesic effects produced by nrTMS were compared with those by non-navigated rTMS, and they found that there was a prolonged effect after stimulating the painful limb’s cortical motor representation by nrTMS [71].

### Limitation of nTMS

It should be noted that nTMS can produce mapping of cortical areas that are close to the surface and therefore reachable by the magnetic field. This signifies that mapping frontobasal and temporomesial gyri are not available, and similar to that, the mapping of the brain region covered by big meningiomas or big arachnoid cysts is unavailable.

## Conclusions

NTMS is the latest technology in the functional localization of the cerebral cortex, which plays an important role in surgical planning, patient consultation, and risk assessment. The results show that the accuracy of nTMS in locating motor and language functions is similar to that of DES, higher than that of fMRI, and it is easy to operate. Therefore, it is recommended that nTMS can be used routinely to locate functional areas in patients with occupied functional areas. NTMS is a useful supplement for established IOM workflows while planning and conducting surgery in suspected motor eloquent regions. NTMS has high application value in the treatment of neurological and psychiatric diseases.

## Abbreviations

3D: Three dimension
ALS: Amyotrophic lateral sclerosis
CSE: Cortical spinal excitability
CST: Cortical spinal tract
DES: Direct electrical cortical stimulation
DTI-FT: Diffusion tensor imaging fiber tracking
EMG: Electromyogram
En-TMS: Electric-field navigated transcranial magnetic stimulation
fMRI: Function magnetic resonance imaging
IOM: Interoperative monitoring
Ln-TMS: Line-navigated transcranial magnetic stimulation
MEP: Motor-evoked potential
MRI: Magnetic resonance imaging
nTMS: Navigated transcranial magnetic stimulation
RMT: Resting motor threshold
ROI: Regions of interest
rTMS: Repetitive transcranial magnetic stimulation
TMS: Transcranial magnetic stimulation
